# Non-Mammalian Eukaryotic Expression Systems Yeast and Fungi in the Production of Biologics

**DOI:** 10.3390/jof8111179

**Published:** 2022-11-08

**Authors:** Mary Garvey

**Affiliations:** 1 Department of Life Science, Atlantic Technological University, F91 YW50 Sligo, Ireland; mary.garvey@atu.ie; Tel.: +353-071-9305529; 2 Centre for Precision Engineering, Materials and Manufacturing Research (PEM), Atlantic Technological University, F91 YW50 Sligo, Ireland

**Keywords:** biologics, eukaryotic, expression systems, glycosylation, monoclonal antibodies

## Abstract

Biologics have become an important area of medical research generating therapeutics essential for the treatment of many disease states. Biologics are defined as biologically active compounds manufactured by living cells or through biological processes termed bioprocessing. Compared to small molecules which are chemically synthesised they are relatively complex and therapeutically specific molecules. Biologics include hormones, vaccines, blood products, monoclonal antibodies, recombinant therapeutic proteins, enzymes, gene and cellular therapies amongst others. For biologic production prokaryotic and eukaryotic cells (mammalian and non-mammalian) are used as expression systems. Eukaryotic expression systems offer many advantages over prokaryotic based systems. The manufacture of high-quality proteins for human clinical use via recombinant technologies has been achieved in yeast and filamentous fungal systems. Advances in bioprocessing such as genetic engineering, bioreactor design, continuous processing, and quality by design has allowed for increased productivity and higher yield in in these non-mammalian eukaryotic systems with protein translation similar to mammalian systems. The application of eukaryotic expressions systems for the manufacture of biologics of therapeutic importance are described herein.

## 1. Introduction

The manufacture of biologics is a rapidly growing industry as these specific therapeutics offer targeted treatment approach for many chronic and prevalent medical conditions including cancer, cardiac disease, neurological disease and autoimmunity. According to the Food and Drug Administration (FDA), a biologic is a therapeutic substance produced by a biological process using biological systems as opposed to the process of chemical synthesis (small molecules) and includes vaccines, antibody therapies, non-vaccine therapeutic immunotherapies, gene therapies and cell therapy [1]. While both production systems have definitive advantages (Table 1), biologics differ from synthetic small molecule drugs in terms of cost, production, administration, and clinical efficacy. The use of biotechnology and recombinant technology to manufacture therapeutic biologics relies on the use of living systems, molecular engineering and bioreactors (typically submerged state fermentations) to produce large molecules displaying desired biological activity. Living systems in use as biologic production platforms at industrial scale include prokaryotic bacterial species, e.g., *Escherichia coli*, eukaryotic yeast and fungal systems, e.g., *Saccharomyces cerevisiae*, *Aspergillus*, plant systems, insect systems, mammalian and human expression systems and cell lines [2]. Currently, small molecules account for 90% of global therapeutic sales as they are used for the treatment of chronic conditions, the biologics market however, is increasing [3]. The biologics market is predicted to reach $580.5 billion (EUR 513.5 billion) by 2026, from a cumulative sales value of $652 billion from 2014 to 2017 [4]. Indeed, Recombinant DNA (RDNA) technology has enabled the production of many biologically active proteins used in disease prevention, treatment and management [4]. The biologic Humira (by AbbVie), a recombinant monoclonal antibody (Mab) used to treat autoimmune disease is currently the highest selling therapeutic globally, generating 60% of AbbVie’s revenue [5]. In cancer therapy, biologics such as Herceptin offer treatment options currently unmet as potent anticancer agents in therapeutic cocktails [6]. More recently, Chimeric antigen receptor (CAR) T cell therapy has emerged as a game changer in cancer treatment. CAR T cell therapy is based on genetically engineering patient T cells to selectively attack cancer cells expressing a specific target antigen [7]. Recently, advances are also being made in the application of Cell-free systems for the production of biologics without using living cells [4].

Bioprocessing occurs in 4 phases: strain/cell line selection and propagation, upstream processing (fermentation), downstream processing, and drug formulation with one biologic usually produced from 1 cell strain [8]. The bioprocessing systems in use for the production of many biologics typically use mammalian Chinese hamster ovary (CHO) cells and murine myeloma cells, with a recent shift towards the use of human derived cell lines [2] due to the ease of post translational modifications (PTMs). PTMs play a vital role in biological processes functioning in many molecular pathways, where PTM errors are observed in many disease states [9]. Fungal and yeast cell systems however, offer many advantages as expressions systems for numerous biologic types. Yeast expressions systems are robust, amenable to genetic engineering or genetic modification (GM), cost-effective, possess native PTM machinery, and do not release endotoxins during processing [10]. Indeed, yeast demonstrate prokaryotic (rapid cell division, single cells, ease of growth) and eukaryotic features (cell organelle, PTM activity) simultaneously making them ideal candidates in the manufacture of recombinant proteins [11]. Features including low production cost, high titre value, pyrogen free, and current classification as Generally Recognised As Safe (GRAS) organisms [10]. Fungal strains of species including *Aspergillus* and *Penicillium* are considered as GRAS by the FDA and are used as expression systems by many biotechnology companies to produce varied biological products [12]. In contrast, to the unicellular yeast systems however, filamentous fungi have complex morphological features in submerged cultures which can be challenging for industrial scale up [13]. This review outlines the application of yeast and fungal cells as platforms for the production of biologics.

## 2. Application of Non-Mammalian Eukaryotic Cells in Bioprocessing

Eukaryotic cell lines, including CHO cells, human cells and insect cells, are invaluable expression systems for the production of many recombinant proteins [14]. Mammalian cell lines of animal and human origin however, are costly and prone to microbial contamination issues with viral species representing the greatest treat [15]. With advances in recombinant protein technology, expression of recombinant protein-based biopharmaceuticals in prokaryotic and non-mammalian eukaryotic cells has become cheaper, more productive, promoting the industrial production of many biologics at industrial scale. Unlike eukaryotic systems, prokaryotic expression systems often produce proteins which do not fold properly, are inactive, produce endotoxins and proteins not amenable to PTM [16]. PTM involves any process which alters the protein composition and includes the irreversible or reversible addition of a chemical group, e.g., phosphate, carbohydrates termed glycosylation, and polypeptides in ubiquitylation [9]. Such alterations are often related to the biological activity of the protein due to improper folding and its direction within the cells, where a loss of functionality may occur [17]. Glycosylation in particular is of significance, as ca. 60% of protein biologics are therapeutic glycoproteins [18]. It is also noteworthy that the over glycosylation of proteins can negatively impact enzyme activity, including enzyme binding and protein stability [19]. PTMs takes place in several cell organelles including the nucleus, cytoplasm, endoplasmic reticulum (ER) and Golgi apparatus [9]. Proteins produced via prokaryotic expressions systems therefore, must pass through an in vitro process for the insertion of PTM adding steps during the synthesis, increasing costs and reducing yield [18]. Additionally, yeasts have a high robustness and tolerance of the harsh fermentation conditions present in bioreactors and bioprocessing scale up [20]. Non-mammalian eukaryotic systems (yeast and fungi) therefore, have clear advantages over prokaryotic systems (Table 2).

### 2.1. Yeast Cell Systems in Biologics Manufacturing

Yeast are single celled microorganisms within the Fungus kingdom, being defined as unicellular fungi. Yeast are eukaryotic microbial species having a cell wall and membrane bound organelle unlike bacteria being prokaryotic. Fungi growing as a yeast morphology have historically been used in the production of food and beverages [25]. Due to their eukaryotic nature, yeast have also long been established as models for the study of mammalian cells, biochemical pathways and evolution. As host expression systems yeast have the advantages of rapid growth, high cell density, relatively inexpensive media requirements, and ease of genetic manipulation found in bacteria coupled with the ability of post-translational modifications, such as proteolytic processing, folding, disulfide bond formation and glycosylation observed in mammalian cells [18]. Yeast commonly conduct modifications including acetylation, amidation, hydroxylation, methylation, N-linked glycosylation, O-linked glycosylation, phosphorylation, pyrrolidone carboxylic acid, sulfation, and ubiquitylation similar to the PTMs of mammalian cells [26]. Glycosylation however, remains a challenge in recombinant protein production from yeast as glycosylation differs from human N- and O-glycosylation [18]. Efforts to improve glycosylation in yeast and improve protein folding and stability include glycoengineering, improving secretory machinery and protein degradation in vivo [10]. As such, yeast are an excellent choice for the industrial production of recombinant therapeutic proteins. Traditionally, *Saccharomyces cerevisiae* is used for recombinant therapeutic protein production having been applied in the production of hormones glucagon and insulin and Hepatitis B vaccine, at industrial scale [17]. Indeed, the expression of Hep B surface antigens led to the development of the first recombinant vaccines using *S. cerevisiae* [27]. Currently, this species and *Pichia pastoris* (renamed to Komagataella phaffii) are the expression systems used in vaccine development, protozoal proteins and tumour antigens [27]. *S. cerevisiae* also displays a broad range of pH tolerance and elevated osmotic pressure [21]. Production of recombinant proteins in *S. cerevisiae* can be done using 3 types of vectors: integration plasmids (YIp), episomal plasmids (YEp), and centromeric plasmids (YCp) [18]. *P. pastoris* is also amenable to bioengineering where genetically engineered strains are able to produce heterologous biologics with human glycosylation profiles [8]. *P. pastoris* produces a higher protein yield than *S. cerevisiae* due to its biochemical processes relating to a lack of ethanol production under aerobic conditions allowing for a higher biomass and protein production [18]. While *P. pastoris* favours respiration over fermentation, *S. cerevisiae* produces ethanol under aerobic conditions with glucose as a food source reducing protein yield, termed the Crabtree effect [22]. *P. pastoris* is currently used in the production of interleukin 1-β for the treatment of autoimmune disease, interferon-α for the treatment of hepatitis B and C and cancer and macrophage colony-stimulating factor (M-CSF) for the treatment of hematopoietic disorders [22]. *P. pastoris* has also been implemented in the production of human insulin, human serum albumin, hepatitis B vaccine, trypsin, and collagen, among others [18]. *P. pastoris* however, remains a non-conventional yeast where the genome has not been fully elucidated which hinders genetic engineering unlike the well-established conventional *S. cerevisiae* [18]. *P. pastoris* can efficiently produce and secrete fully active heterologous proteins, e.g., pochymosin, poorly secreted by *S. cerevisiae* [23]. The ability of yeast to secrete protein products has many advantages in the production of biologics including easier isolation, purification, no toxic intracellular build-up of heterologous protein therefore, reducing production costs [20]. The non-conventional yeast *Yarrowia lipolytica* is recognised for its ability to produce heterologous recombinant proteins. *Y. lipolytica* is a human commensal, obligate aerobe, GRAS and is capable of folding and secreting large and/or complex heterologous proteins in contrast to *S. cerevisiae* [24]. Indeed, this species is less prone to hyperglycosylation which is a feature of *S. cerevisiae* recombinant proteins [28]. Engineered *Y. lipolytica* strains produce several heterologous valuable metabolites including carotenoids, terpenes, polyketides, aromatic amino-acid-derived molecules and therapeutic biologics interferon α, epidermal growth factor, blood coagulation factor XIIIa, proinsulin and insulinotropin, cytochrome P450 enzymes, and oestrogen receptor α [29]. Currently, ca. 150 recombinant proteins have been produced using *Y. lipolytica* expression systems however, only 25 of these are produced at industrial scale or bioreactor scale despite this species having similar yield and productivity as *S. cerevisiae* and *P. pastoris* [30]. Scale up is hindered by issues relating to metabolic load, unpredictable dimorphism and oxygen needs of the strain [30]. The non-conventional yeast *Ogataea polymorpha* (formerly *Hansenula polymorpha*) has been used as an expression system for the production of hepatitis B vaccines and insulin-like growth factors [31]. *H. polymorpha* is thermotolerant with a temperature range of 30 to 50 °C allowing proteins with biological activity at 37 °C as obtained with other yeast including *S. cerevisiae* with PTM and reduced hyperglycosylation [32].

#### 2.1.1. Polyketides and Non-Ribosomal Peptides

Polyketides and non-ribosomal peptides constitute a group of small molecules having complex chemical structures produced by microbial species, plants and marine organisms which allow for environmental adaption, communication between species and self-protection [27]. Polyketides and non-ribosomal peptides are biosynthesized by the enzymes polyketide synthases (PKSs) and non-ribosomal peptide synthetases (NRPSs), respectively [33]. Many of these molecules or their hybrids are of clinical relevance as biologic therapeutics as they display anticancer activity, e.g., calicheamicin, immunosuppressive action, e.g., rapamycin and antibacterial action, e.g., vancomycin [34]. Recently, the application of yeast in the production of these biologics via enzymatic biochemical pathways has gained momentum. The anti-cholesterol drug Lovastatin for example is typically produced by the species *Aspergillus terreus* a filamentous fungus, where yeast *S. cerevisiae* and *P. pastoris* may offer a more favourable non-pathogenic production system [19].

#### 2.1.2. Vaccine Production

Recent advances have moved towards protein-based vaccines, virus-like particle vaccines, viral vector and nucleic acid-based vaccine production in an attempt to mitigate the time constraints, pathogenicity, immunogenicity and biocompatibility issues observed with traditional vaccine types [10]. Recombinant technology and microbial expression systems are used in the production of subunit-based and viral like particle-based vaccines [35]. Indeed, yeast platforms and the production of whole yeast-based vaccines (WYVs) has appeared for combating infectious disease and treatment of cancers [10]. The non-pathogenic *S. cerevisiae* offers many advantages in the production of vaccines including strong adjuvant properties, long term antigen stability, ease of GM and ability to survive the gastrointestinal tract allowing for oral administration [36]. Studies have shown that whole recombinant *S. cerevisiae* cells expressing foreign antigens can activate dendritic cells (DCs), robust antigen-specific cytotoxic T lymphocyte (CTL) responses, and confer protective cell-mediated immunity in animal studies regardless of yeast cell viability [37]. WYVs based on yeast cells expressing specific tumour antigens offers potential immunotherapy for the treatment of cancer, e.g., melanoma, papilloma, leukemia and carcinoma [23].

#### 2.1.3. Monoclonal Antibodies

Monoclonal antibodies (mAb) have many therapeutic applications in the treatment of infectious disease, inflammation and cancer where their glycosylation is key to biologically [38]. The production of mAb in yeast expression systems is hindered due to incompatible surface glycosylation, therefore, few antibody molecules have been functionally expressed in yeast systems [20] where CHOs cells remain the basis of mAb production at industrial scale. The glycosylation of mAbs in the Fc region impacts on the interaction of the antibodies with effector cells of the immune system and biological activity [38]. The hyper-mannose glycosylation of mAbs caused by *S. cerevisiae* results in an immunogenic reaction in humans [10]. *P. pastoris* is less prone to this mannose hyperglycosylation [18]. Experimental studies using targeted genetic engineering to improve the glycosylation of yeast produced mAb have been described [38]. Genetically modifying single genes, genes families or the whole genome has been made possible using CRISPR-cas9 technology [39]. Nonetheless, mAbs as biologics is hindered by their limited tumour penetration, high manufacturing costs, immunogenicity and potential therapeutic resistance [40]. Therapy based on polyclonal antibodies (pAbs) which are a mix of synergistic mAb having action on multiple epitopes, offer some advantages. pAbs such as ZMapp for Ebola treatment combines 3 mAb and the anticancer pAb combination of lumiliximab and rituximab displays increased antitumour efficacy [8]. Biologics termed minimal antibody-binding fragments including antigen-binding fragment (Fab), single-chain variable fragment (scFv) and single V-type domain have many advantages such as high specificity, high affinity, enhanced tissue penetrability, stability, solubility, reduced immunogenicity, and cheaper industrial scale production [20]. RDNA tech and the use of microbial expression systems offers a means of producing large scale specific antibody fragments or recombinant antibody fragments (rAb) which are easier to isolate than those produced in mammalian cell culture systems. Prokaryotes *E. coli* and *Bacillus subtillis* and yeast *S. cerevisiae* and *P. pastoris* have been used to express rAbs [41]. In order to produce the high yield required for therapeutic use obstacles such as time of production and optimal cryopreservation protocols need to investigated [41]. Antibodies fragments as therapeutic biologics are limited by a short serum half-life and aggregation-induced immunogenicity [40]. Antibodies fragments are commonly used in engineered yeast surface display (YSD) technologies where a genetic fusion of the antibodies fragment to the yeast cell surface antibodies fragment is performed [42]. The PD-1 blocking antibody Sintilimab generated using yeast display technology gained approval for the treatment of Hodgkins lymphoma [43]. The discovery of heavy-chain only antibodies (HcAbs) termed nanobodies in camelids has led to the latest antibody driven research particularly in cancer diagnosis and treatment [40]. Nanobodies are robust and relatively amenable to large scale production, low immunogenicity, have the ability to display diverse anti-tumour targets makes them desirable for therapeutic use [40]. Studies describe the use of an engineered *S. cerevisiae* as an in vitro nanobody platform [44].

### 2.2. Fungal Cell Systems in Biologics Manufacturing

Fungal species have a long history in the food and beverage industries; however, many enzymes, organic acids, antimicrobial peptides and antibiotics are also produced by filamentous fungi. The filamentous fungi including moulds and mushrooms having extremely diverse metabolic capabilities producing organic acids, antibiotics, proteins, enzymes, vitamins, fatty acids amongst other compounds leading to their application in many industries and more recently as meat alternatives and vegan leather products [25]. As production systems for biologics filamentous fungi have advantages over yeast as they have powerful secretory pathways and perform PTM of eukaryotic proteins like mammalian cells [45]. Indeed, filamentous fungi can conduct PTM including glycosylation, peptide chain shearing, and disulfide bond formation, similar to those of mammal cells [19]. Over glycosylation is also less extensive in fungi than in yeast including *S. cerevisiae*. Additionally, fungi expression systems are a cheap fermentation process, where the extracellular proteins are easier purified compared to intracellular proteins [45].

While GM is more difficult in fungi and the development of filamentous fungal expression platforms is much more complex and time-consuming, the protein yield is 10–1000-fold that of yeast or mammalian cells making them worthy of development [46]. For recombinant production of biologics, the most commonly used fungi are the GRAS strains of *Aspergillus niger*, *Aspergillus oryzae*, *Trichoderma reesei* and *Neurospora crassa* being well characterized by whole-genome-sequencing, transcriptomics, gene annotations, and genetic engineering tools including adaptive laboratory evolution, and radiation [47]. Unlike yeast, fungi present with highly complex morphological features in submerged cultures where their growth morphology can range from fully dispersed mycelium to compact pellet structure [13]. Productivity and yield are affected by slight changes in fungal morphology affecting mass transfer and mixing within the bioreactor environment. Genetic analysis and editing may offer mechanisms of controlling morphology, protein and metabolite secretion to allow for industrial scale up [47].

Fungal bioprocessing is typically performed in either submerged fermentation bioreactors using free flowing liquids or solid-state fermentation systems (SSF) better mimicking the natural growth patterns of filamentous fungi [45] or as biofilms attached to a surface. Submerged fermentation is the most widely used mode of fermentation [48]. SSF however, has scale up limitations including contamination issues, low substate utilisation rate and a lack of commercial SSF reactor designs [49]. SSF has become a sought-after system in bioprocessing as it holds many advantages including high productivity, less waste, lower energy needs, and lower contamination risk [50] making it a more environmental green process. Oxygen transfer, temperature, pH and water regulation are key limiting factors in the bioreactor design of SSFs with fungal morphology also impacting operation [48].

Filamentous fungi produce a vast array of molecules (Table 3) which may have biological therapeutic potential including taxol an anticancer drug produced by endophytic fungi *Fusarium oxysporum* and *A. niger* [51]. To date, taxol has not been produced by filamentous fungi at industrial scale relating to issues of specific growth requirements as an endophytic organism where light exposure may affect taxol synthesis [52]. Asparaginase an enzyme used for the treatment of hemotopoeitic disease including acute lymphoblastic leukemia and non-Hodgkin lymphoma is produced by *Mucor hiemalis*, *A. niger*, *A. flavus*, *A. nidulans*, *A. terreus* [53]. *Trichoderma* has been cultivated using submerged, solid-state and biofilm fermentation to produce the secondary metabolite and immunosuppressant cyclosporin [48]. Optimising filamentous fungi for the industrial production of asparaginase may offer a non-immunogenic biologic to replace that which is currently produced in *E. coli* expression systems where death is a risk to patients [54]. Filamentous fungi produce a wide range of proteins and enzymes meaning that protein purification at downstream processing may be complicated with increasing costs at industrial scale [55]. Additionally, biological compounds in higher fungi (mushrooms) are structural similar to endogenous neurotransmitters and can act agonists of neurological pathways potentially offering treatment of psychiatric conditions [56].

## 3. Industrial Considerations

The first step in yeast recombinant protein production is construction and genetic engineering of the strain for scale up in bioreactors [11]. The GM of host cells for heterologous protein production involves the over expression of genes and some deletion of host genes via the use of vectors (expression vectors) or plasmids which deliver the gene of interest to the host cells [28]. Heterologous DNA is either episomal maintained as a plasmid or integrated into the host chromosomal DNA with the latter being more frequent in yeast species [28]. Eukaryotic genetic material consists of protein coding regions termed exons surrounded by non-coding regions termed introns, unlike prokaryotes which do not contain introns [59]. Isolating out the gene of interesting (coding a biological active protein) means splicing introns to generate mRNA. For heterologous production in yeast cells an expression cassette containing a promoter, open reading frame containing the protein gene for regulation of expression must be present [28]. While not essential to end transcription a terminator region can also be added. The terminator region functions to end transcription and can influence gene expression levels of the protein and enhance mRNA stability consequently improving protein productivity [31]. The inclusion of an intron in an expression cassette may also enhance its transcription by many folds [30]. Introns significantly enhance the transcriptional output of genes with many human genes including growth hormone needing introns for normal expression [60]. A promoter is a short segment of DNA which acts as the start point for gene transcription by RNA polymerase. In yeast there are 2 promoter types; constitutive promoters and inducible promoters. In bioprocessing well known constitutive and inducible promoters having strong transcriptional action are implemented to achieve overproduction of desired biologics in yeasts [18,61]. Constitutive promoters allow for simplicity and maintainable levels of expression, where inducible promoters are often implemented when separation of growth and production is needed [18]. Inducible promoters can alter their transcriptional activity in response to stimuli including carbon sources and environmental factors [61] allowing for some control over gene expression levels. Inducing protein expression via chemicals or modifying environmental conditions during fermentation in the bioreactor minimises metabolic load and reduces the toxicity impact of recombinant proteins on host cells [62]. Methylotrophic yeast including *P. pastoris* and *H. polymorpha* can use methanol as a carbon source allowing for the use of methanol inducible promoters [32]. At industrial scale the use of inducible promoters in fed-batch bioreactors enables cell growth at high growth rate prior to protein expression allowing for some influence on metabolic load [30]. The most frequently utilized promoters in yeasts are described by researchers elsewhere [18]. The construction of synthetic promoters allowing for increased protein expression, enhanced protein folding and more regulated transcription is much sought after [30]. A codon is a sequence of 3 nucleotides essential to protein translation where codons encoding the same amino acid are called synonymous codons which is prone to species specific usage bias [63]. Studies have suggested that codon usage is an important determinant of mRNA stability in Saccharomyces cerevisiae as it regulates translation elongation and translational protein folding processes [64]. In yeast expression systems studies are warranted to determine the influence of codon optimization on protein expression [65]. In the design and bioengineering of recombinant yeast, the use of genetic tools and genetic tractability encourages the use of conventional yeast such as *S. cerevisiae*. There is a need to elucidate the genetic profile, metabolic and biochemical systems of the non-conventional yeast in order to better apply strains such as *Y. lipolytica* in bioprocessing where their advantages over *S. cerevisiae* can be harnessed. For example, sequencing of the genome of *Y. lipolytica* has determined that this species shares genomic traits with filamentous fungi where this species is considered a dimorphic yeast [28]. This morphological state of *Y. lipolytica* has not been elucidated in bioreactors where the dimphorism variability impacts on heat and mass transfer in the reactor impacting protein formation [30]. Other non-conventional yeasts include *P. pastoris*, *K. lactis*, *Y. lipolytica*, *H. polymorpha* and *S. pombe* for the synthesis of recombinant proteins [11].

The operation of bioreactors is affected by cell type, media composition, substrate concentration, cell density of the biocatalyst, product inhibition, pH, temperature with productivity increased in continuous mode over batch systems [66]. Optimising media composition, temperature, pH, cell growth and protein production kinetics aids in increasing recombinant protein production by yeast cells. Importantly, a shortage of specific amino acids and energy shortages can lead to translational errors in the recombinant protein impacting protein stability and immunogenicity [30]. Heterologous proteins produced in the host cell may be prone to proteolytic degradation as they are foreign proteins present in the cell [62]. Genetically engineering protease deficient yeast strains aids in overcoming this limitation [11]. Increasing cell density increases product yield, where retaining cells inside the bioreactor aids in reducing costs and improving cell density. Dissolved oxygen (DO) is an important consideration in bioreactor set up as DO impacts aerobic cell growth kinetics, cell physiology and stability, with increasing requirements during exponential phase where cells are dividing and metabolically active [30]. Fed-batch and continuous reactor cell density is complicated by processes requiring varying types of equipment for set up, have a high operational cost, long cultivation time and long periods required for downstream processes and are prone to the Crabtree effect [67]. In a continuous system cell retention is accomplished via filtration of the product through a membrane, immobilization on a carrier material or by exploiting cellular flocculation [66]. Expression of the heterologous protein causes cell resources to be applied to the transcription and translation of the recombinant protein at the expense of the host cell metabolism. The term metabolic load describes this allocation of resources to heterogenous protein production at the cost of cellular activity to the detriment of the cell, reduced growth capacity and specific growth rate [30]. Furthermore, the effect of metabolic load is greater with larger recombinant gene sizes, copy number, expression levels and issues with nutrient and dissolved oxygen availability [68]. Secretion of the desired protein product greatly simplifies the extraction and purification processes of downstream processing [28]. Protein secretion is initiated by transporting the protein through the membrane of endoplasmic reticulum (ER) in response to signals which directly impact the protein yield [11]. Misfolded protein is degraded prior to secretion and may damage the ER, the presence of protein folding chaperones and redox enzymes should be over expressed in yeast systems [11]. Studies describe the production of more than one drug or combination drugs simultaneously in a single batch which offers clear advantages over single biological production [8]. Synthetic biology as recently emerged as a promising field in the area of medical and therapeutic science having application in the production of drugs, vaccines and biosensors [69]. Synthetic biology involves the assembly of a specialized systems designed for a biologic purpose and incorporates engineering into bioprocessing technologies. As expression systems for synthetic biology *E. coli* and *S. cerevisiae* are most frequently utilised due to their broad ranging advantages [70]. Synthetic biology allows for enhanced production of biologics via modulation of gene circuits impacting on expression levels, protein levels, and pathway levels of expression systems via engineering of promoters, terminators, etc. [71].

## 4. Conclusions

For the production of biologics yeast are classified as either non-methylotroph and methylotroph species. At industrial scale *S. cerevisiae*, a non-methylotroph, *P. pastoris,* and Hansenula polymorpha being methylotrophic yeasts are implemented for recombinant protein production. Yeast such as *Komagataella* sp., *Kluyveromyces lactis*, and *Y. lipolytica* have also emerged as advantageous expression systems. As non-mammalian eukaryotic expression systems, *S. cerevisiae* is a preferred production system for a variety of biologics due to its innate ability to secret proteins, is toxin free, good expression levels, and ability to perform PTMs. PTMs relates to protein biological activity as it impacts on protein folding where misfolded proteins lose activity and stability. While glycosylation in yeast is different than in mammalian cells, the use of RDNA technology has allowed for genetically engineered strains enabling some humanization of yeast glycosylation. *S. cerevisiae* has also some important disadvantages as this species is not suitable for high-density culture, regulation promoter deficits, and low secretion of proteins greater than 30 kDa. Such limitations have encouraged the application of non-conventional yeasts including *P. pastoris* as expression systems. Filamentous fungi as expression systems for biologics offers many advantages including glycosylation and other PTMs more similar to human proteins, high density growth adaptability and a high rate of protein expression with low media requirements. Indeed, the secretory potential of filamentous fungi, e.g., *Aspergillus* sp. and *Trichoderma* sp. is described as being ten times higher than that of *S. cerevisiae*. The use of non-mammalian eukaryotic cells also contributes to more environmentally friendly industrial production processes requiring less energy input and generating less waste than mammalian systems. The use of yeast expression systems to produce recombinant protein, virus-like particles, and yeast surface display for development as oral vaccines offers a new strategy for vaccine administration. Optimising eurkaryotic production platforms may provide increased productivity of key therapeutics in the near future. Due to their high-cost comparative to small molecule therapy biologics are relatively inaccessible to many patients and so are not chosen for treatment. Reducing production costs may allow for increased accessibility to these potent therapeutics allowing for better access and improved health care globally.

## Figures and Tables

**Table 1:** Outlining the advantages of traditional chemical synthesis of small molecules and the advantages of biologics.

Small Molecule Drugs	Biologics
Predictable pharmacokinetics and pharmacodynamics profiles [6]	Biologic proteins are highly specific and potent with extended effect [11]
Easier manufacturing, characterizing, and regulatory processes [6]	Biologics are less toxic
Cheaper for consumers, cheaper to manufacture	Targeted treatment, e.g., CAR T cell therapy [7]
Oral bioavailability and stability	Can produce large peptides and proteins [4]
Generic versions available	Applicable to real time process control
Do not suffer microbial contamination issues to the same extent	Many biologics are losing their patent protection and other exclusivity rights leading to production of biosimilars [4]

**Table 2:** Outlining the advantages and limitations of yeast expression systems in the production of biologics.

Advantages	Limitations
Less susceptibility to contaminations by phages [11]	Limited glycosylation capacity compared to mammalian cells
Scalable at industrial level	Difficulty in cell disruption [16]
Existing regulatory approval [16]	Proteins denaturation, temperature changes, pH, organic solvents presence, surface and interface interactions that can form protein aggregates at [21]
More improved secretion efficiency than bacteria [11]	Ethanol production by S. cerevisiae (Crabtree effect) [22]
Yeast has tolerance to low Ph and fermentation inhibitors and harsh fermentation conditions [20]	Extracellular excretion is not large [22]
Yeast efficiently modifies its recombinant proteins post transnationally	Yeast glycosylation is dissimilar to mammalian glycosylation (high mannose type) [10]
Considered GRAS [10]	Heterologous proteins expressed in *S. cerevisiae* are hyperglycosylated [23]
More adaptable to harsh industrial scale up [18]	
Yeast based vaccines which are edible, economic and induce immune response [23]	
*Y. lipolytica* strains biosynthesis of metallic nanoparticles for biomedical applications [24]	

**Table 3:** Biologically active compounds produced by filamentous fungi.

Biologic/Therapeutic	Filamentous Fungi	Disease Treatment
Paclitaxel (Taxol)	*Taxomyces andrenae*, *F. oxysporum* and *A. niger*	Cancer [51]
Beta-galactosidase	*A. foetidus*	Lactose intolerance, GM1-gangliosidosis [57]
Lovastatin (statin)	*Monascus ruber*, *A. terreus*	high blood cholesterol and reduce the risk of cardiovascular disease
l-asparaginase	*M.hiemalis*, *A.niger*, *A. flavus*, *A. nidulans*, *A. terreus* [53]	Acute lymphoblastic leukemia and non-Hodgkin lymphoma
Ergot alkaloids	*Claviceps purpurea*	Parkinson’s disease, cluster headaches and migraine [56]
Hericenones and erinacines (cyathane derivatives)	*Hericium erinaceus*	Alzheimer’s and Parkinson’s disease [56]
Proteases/proteinases/peptidases	*Aspergillus* and *Penicillium species*	Cardiovascular disease, emerging agents in the treatment of sepsis, digestive disorders, inflammation, cystic fibrosis, retinal disorders, psoriasis [58]
Amphotericin B (AMP B)	*Penicillium nalgiovense*	Antifungal, treatment of invasive fungal infections mucormycosis, aspergillosis, blastomycosis, candidiasis, coccidioidomycosis, and cryptococcosis [51]
Griseofulvin	*P. griseofulvum*	Antifungal, treatment of dermatophytoses

## Data Availability

Not applicable.
